# PERSPECTIVES ON HEALTH AND AGING WITH SPINAL CORD INJURY: A QUALITATIVE DESCRIPTIVE STUDY

**DOI:** 10.1093/geroni/igac059.063

**Published:** 2022-12-20

**Authors:** Laura Baehr, Kathleen Fisher, Margaret Finley

**Affiliations:** Drexel University, Philadelphia, Pennsylvania, United States; Drexel University, Philadelphia, Pennsylvania, United States; Drexel Univeresity, Philadelphia, Pennsylvania, United States

## Abstract

Age of spinal cord injury (SCI) onset and life expectancy have increased over the last half century, but minimal inquiry on health and aging lived experience perspectives are available. The objective of this study was to begin to examine this through a qualitative descriptive design. Nine group interviews were conducted with individuals living with SCI (n=24) 22-76 with injury duration 3-47 years. Participant descriptions on health and aging were thematically analyzed. Health maintenance was related to physical routine to prevent secondary health conditions, injury acceptance, and engagement with disability networks. Aging outlook was connected to fear of dependence and lack of education on aging with SCI. These findings demonstrate that clinicians and researchers should investigate issues beyond routine self-management to support life with SCI. Personal considerations based on life stage when injured warrants investigation. Advocacy for peer-support is imperative at all life stages given its positive impact on health.

